# DNA-Demethylase Regulated Genes Show Methylation-Independent Spatiotemporal Expression Patterns

**DOI:** 10.3389/fpls.2017.01449

**Published:** 2017-08-28

**Authors:** Ulrike Schumann, Joanne Lee, Kemal Kazan, Michael Ayliffe, Ming-Bo Wang

**Affiliations:** ^1^CSIRO Agriculture and Food, Canberra ACT, Australia; ^2^CSIRO Agriculture and Food, Queensland Bioscience Precinct, St. Lucia QLD, Australia

**Keywords:** DNA-demethylation, ROS1, Demeter-like, *Fusarium*, *Arabidopsis thaliana*

## Abstract

Recent research has indicated that a subset of defense-related genes is downregulated in the Arabidopsis DNA demethylase triple mutant *rdd* (*ros1 dml2 dml3*) resulting in increased susceptibility to the fungal pathogen *Fusarium oxysporum*. In *rdd* plants these downregulated genes contain hypermethylated transposable element sequences (TE) in their promoters, suggesting that this methylation represses gene expression in the mutant and that these sequences are actively demethylated in wild-type plants to maintain gene expression. In this study, the tissue-specific and pathogen-inducible expression patterns of *rdd*-downregulated genes were investigated and the individual role of *ROS1, DML2*, and *DML3* demethylases in these spatiotemporal regulation patterns was determined. Large differences in defense gene expression were observed between pathogen-infected and uninfected tissues and between root and shoot tissues in both WT and *rdd* plants, however, only subtle changes in promoter TE methylation patterns occurred. Therefore, while TE hypermethylation caused decreased gene expression in *rdd* plants it did not dramatically effect spatiotemporal gene regulation, suggesting that this latter regulation is largely methylation independent. Analysis of *ros1-3, dml2-1*, and *dml3-1* single gene mutant lines showed that promoter TE hypermethylation and defense-related gene repression was predominantly, but not exclusively, due to loss of ROS1 activity. These data demonstrate that DNA demethylation of TE sequences, largely by ROS1, promotes defense-related gene expression but does not control spatiotemporal expression in Arabidopsis.

**Summary**: *Ros1*-mediated DNA demethylation of promoter transposable elements is essential for activation of defense-related gene expression in response to fungal infection in *Arabidopsis thaliana*.

## Introduction

Epigenetic modifications, such as DNA cytosine methylation, have recently been implicated in plant defense against bacterial and fungal pathogens (Zhu et al., 2016). In plants, DNA methylation occurs in all cytosine contexts (CG, CHG, and CHH; H stands for A, C, or T) and involves both *de novo* DNA methylation and maintenance DNA methylation. *De novo* DNA methylation is mediated by RNA-directed DNA methylation (RdDM), a plant-specific RNA silencing pathway directed by 24-nt small interfering RNAs (siRNA) (Matzke and Mosher, 2014). Maintenance methylation of CG and CHG contexts occurs during DNA replication and is catalyzed by Methyltransferase 1 (MET1) and Chromomethylase 3 (CMT3), respectively. Maintenance of CHH methylation generally requires the continuous presence of 24-nt siRNAs and RdDM, although CHH methylation in long transposable element (TE) sequences in highly repetitive chromosomal regions can be maintained by CMT2 (Zemach et al., 2013). In plants, DNA methylation primarily targets TEs and repetitive sequences in the genome.

The DNA methylation level of specific subsets of genomic loci in plants is regulated by the DNA glycosylase family of DNA demethylases (Penterman et al., 2007; Lister et al., 2008; Stroud et al., 2013). DNA demethylases remove 5-methylcytosine and replace it with unmethylated cytosine through a base-excision-repair mechanism (Zhu, 2009). The Arabidopsis genome encodes four DNA demethylase genes; Demeter (*dme*), Repressor of silencing 1 (*ros1*)/Demeter-like 1 (*dml1*), *dml2*, and *dml3*. DME is required for the expression of specific imprinted maternal alleles during seed development (Bauer and Fischer, 2011), while ROS1 is required to maintain the activity of some transgenes and transposons (Zhu, 2009). ROS1 has also been implicated in plant developmental regulation and biotic and abiotic stress responses (Yu et al., 2013; Yamamuro et al., 2014; Bharti et al., 2015). The biological function of DML2 and DML3 remains unknown.

Recent studies using the Arabidopsis–*Pseudomonas syringae* pathosystem have shown that DNA demethylation plays an important role in pathogen defense. For example the DNA demethylase-deficient mutant *ros1* shows increased susceptibility to *P. syringae* infection while *de novo* and maintenance DNA methylation mutants such as *nrpe1, ddc* (*drm1 drm2 cmt3*) and *met1* display enhanced resistance to *P. syringae* (Lopez et al., 2011; Dowen et al., 2012; Yu et al., 2013). Using an Arabidopsis–*Fusarium oxysporum* pathosystem, we recently demonstrated that the triple DNA demethylase mutant *rdd* (*ros1 dml2 dml3)* is highly susceptible to this fungal pathogen when compared with wild-type (WT) plants (Le et al., 2014). Collectively these data demonstrate that DNA demethylation plays a role in both bacterial and fungal disease resistance.

How DNA demethylation facilitates plant defense remains unclear. In our previous study using whole Arabidopsis plant tissues, we found 279 genes to be downregulated in the *rdd* mutant compared with WT plants. A significant proportion of these genes have a role in stress responses and are induced upon *Fusarium* infection (Le et al., 2014). These data suggest that DNA demethylases play a role in plant defense by positively regulating the expression of stress response genes. Most of the *rdd*-downregulated stress response genes contain short TE sequences in their predicted promoter regions which had altered localized methylation changes at some CHH and CG sites in the *rdd* mutant (Le et al., 2014). Methylation of these sites therefore appears to play a regulatory role in the expression of these stress response genes.

Here, we extend our previous study by examining the role of DNA demethylases in regulating tissue-specific and *F. oxysporum*-inducible expression patterns of the *rdd*-downregulated stress response genes. *F. oxysporum* is a vascular pathogen that enters the plant mainly through the roots and colonizes the vascular system (Czymmek et al., 2007; Islam et al., 2012). Expression patterns of these *rdd*-regulated genes were compared in roots and shoots, with and without *Fusarium* infection, and the role of DNA methylation in regulating this spatiotemporal expression determined using Arabidopsis demethylase mutants. Furthermore, the role of the individual DNA demethylase genes, *ros1, dml2*, and *dml3* in the regulation of these genes was investigated. Our findings indicate a role for DNA demethylases and promoter TEs in controlling the magnitude of defense gene expression but not spatiotemporal expression.

## Materials and Methods

### Plant Growth Conditions, *Fusarium* Infection, and Fungal Biomass Measurements

Plants used in this study included WT Arabidopsis Col-0, the triple demethylase mutant *rdd* (Col-0 background with *ros1, dml2, dml3*; SALK_045303, SALK_131712, SALK_056440, respectively) (Le et al., 2014) and the single demethylase mutants *ros1-3, dml2-1*, and *dml3-2* (Penterman et al., 2007). Seeds were sterilized and sown on Murashige and Skoog (MS) agar (Murashige and Skoog, 1962) with 3% sucrose, incubated at 4°C for 2 days and then grown at 22°C with a 16 h light/8 h dark photoperiod. Seedlings were transferred to fresh media 1 week after germination.

*Fusarium oxysporum* f. sp. *conglutinans* strain 5176 (obtained from Dr. Roger Shivas, Queensland Department of Primary Industries and Fisheries, Australia) was grown in liquid potato dextrose broth (PDB) at 28°C for at least 2 days and filtered through miracloth. Eighteen day old Arabidopsis plants were *Fusarium-*infected by briefly dipping the roots into 1 × 10^6^ spores/ml, draining excess solution and immediately transferring the plants to sucrose-free MS plates. Mock-infected plants were dipped in water to serve as a stress control. Disease scoring was performed at 8 days post infection (dpi) by counting the number of plants exhibiting the following symptoms: (0) no chlorotic leaves, (1) 1–3 leaves showing chlorosis, (2) 4–6 leaves showing chlorosis, (3) all leaves showing chlorosis, and (4) dead plant. The experiment was performed in duplicates using at least 15 plants per genotype and repeated independently.

**FIGURE 1:**
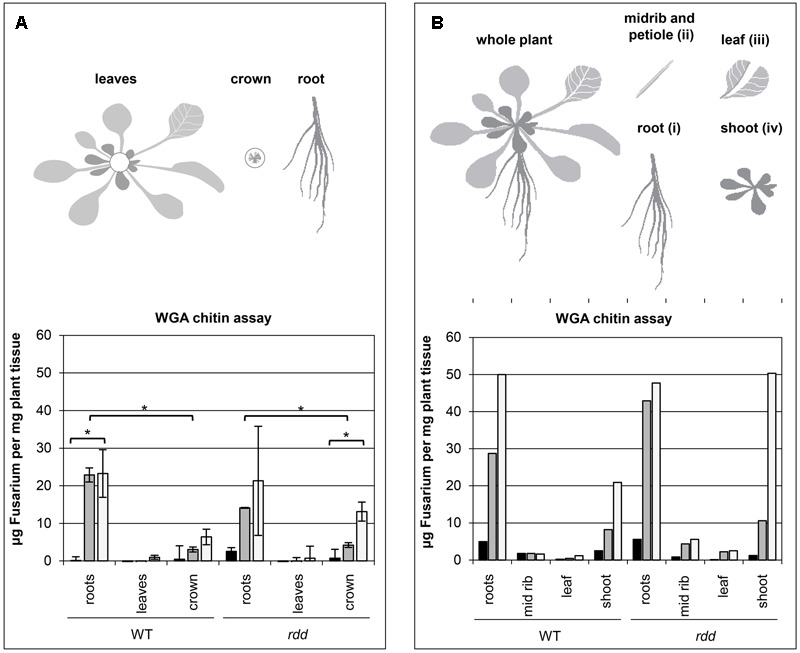
Temporal characteristics of fungal biomass accumulation in Arabidopsis WT and *rdd* mutant plants. **(A)** Top panel depicts a schematic detailing the plant subsections used for the WGA chitin assay. Bottom panel shows the accumulation of fungal biomass. The average of two biological replicates, nine plants per replicate, is shown with error bars indicating standard deviation. Asterisks indicate significance as determined by Student’s *t*-test *p* ≤ 0.05. The temporal characteristics of the *Fusarium*-infected sample are shown by black (1 dpi), gray (3 dpi) and white (6 dpi) bars; mock samples showed negligible fluorescence indicating low background and are omitted here. **(B)** Top panel depicts a schematic detailing the plant subsections used for the WGA chitin assay. Bottom panel shows the accumulation of fungal biomass determined in a single experiment using 12 individual plants per sample. The temporal characteristics of the *Fusarium*-infected sample are shown by black (1 dpi), gray (3 dpi) and white (6 dpi) bars; mock samples showed negligible fluorescence indicating low background and are omitted here.

Plant material was harvested by carefully lifting the plants from the plate and separating root and shoot tissue at the hypocotyl/epicotyl junction. Leaf sections were obtained by cutting individual leaves at the base and then separating the midrib and petiole from the leaf blade (**Figure 1**). Multiple individual plants were pooled for a single biological replicate as indicated. Quantitative measurement of fungal biomass in plant tissue was performed using the wheat germ agglutinin (WGA) chitin assay as described by Ayliffe et al. (2013). Briefly, tissue samples were autoclaved in 1 M KOH, gently neutralized in 50 mM Tris pH 7.0 and then macerated by sonication. A standard curve was created by spiking a known amount of macerated *Fusarium* mycelial tissue into macerated uninfected WT Arabidopsis tissue.

Samples were incubated with a WGA-FITC conjugate antibody that specifically binds to chitin. Excess stain was removed by washing and the remaining fluorescence measured. Measurements were performed in technical quadruplicates. To test whether differential accumulation was significantly different between samples, Student’s *t*-tests were performed between indicated samples.

### RNA Isolation and Quantitative RT-PCR (RT-qPCR)

RNA was either isolated using TRIzol^®^ (Thermo Fischer Scientific) according to manufacturer’s instructions with inclusion of an additional second chloroform extraction, or by hot phenol extraction as described below. RNA samples were treated with RNase-free DNase (Promega) and reverse-transcribed using oligo-dT primers and Superscript III reverse transcriptase (Invitrogen) according to manufacturer’s instructions. RT-qPCR was performed in technical triplicates in the ABI 7900HT Real Time PCR System (Applied Biosystems) using Platinum Taq polymerase (Invitrogen) and SYBR green. Transcript abundance was measured using the standard curve method and normalized to *Actin2* and *FDH* (primers are listed in Supplementary Table S1). Both normalisations gave similar results for all experiments so only the results against *actin2* are shown. All measurements were performed in technical triplicates using biological duplicates. Student’s *t*-tests were performed to test whether differential expression was significant. Samples analyzed are indicated and significance ranges shown. Sample pairs that were not significantly different are not shown to simplify the figures.

### DNA Isolation and Methylation Analysis by Bisulfite Sequencing

DNA (and RNA) was isolated by hot phenol extraction as previously described (Vries et al., 1989) with some modifications. Large RNA was precipitated with LiCl and DNA was precipitated from the supernatant using isopropanol. DNA was further purified by a second ethanol precipitation step. Ten μg of genomic DNA was bisulfite-treated as previously described (Wang et al., 2008). Regions of interest were amplified by nested PCR using the following cycling program: 12 min at 94°C followed by 10 cycles of 1 min at 94°C, 2:30 min at 50°C, 1:30 min at 72°C, and 30 cycles with 1 min at 94°C, 1:30 min at 55°C, 1:30 min at 72°C, with a final extension of 10 min at 72°C (primers were designed as described by Finn et al., 2011; Supplementary Table S1). Products were purified using the PCR clean-up system (Qiagen) and directly subjected to Sanger Sequencing using the BigDye Terminator system (Life Technologies) and the forward primer. Sequencing traces were uploaded into Mutation Surveyor (SoftGenetics, United States) and C to T conversion peak height ratios calculated. Analyses were performed in biological duplicates. To determine the efficiency of bisulfite conversion a region of the chloroplast encoded gene *psaA* was amplified from the bisulfite-treated DNA and sequenced. Conversion for all samples analyzed here was found to be efficient (examples of the traces of *psaA* are shown in Supplementary Figure S1). Significance of differential methylation was determined by unpaired, two-tailed Student’s *t*-test as indicated. Pairings with significant differential DNA methylation levels are indicated in the respective figures. *p*-values are not shown in figures for simplification but can be found in Extended Data Files 1 and 2.

## Results

### The Majority of Fungal Biomass Accumulates in Roots and Meristematic Crown Tissue during *Fusarium* Infection

Previously, we have shown that the expression of a subset of defense-related genes is downregulated at the whole plant level in the Arabidopsis triple DNA demethylase mutant *rdd* upon infection with *F. oxysporum* (Le et al., 2014). Gene expression changes upon pathogen infection can often be localized specifically to infection sites and therefore differ from that of whole plants where the majority of tissues may be uninfected. To further define the regulatory role of DNA demethylation in defense gene expression we first examined the spatial and temporal colonization of Arabidopsis tissue by *F. oxysporum*, which is a poorly characterized process (Czymmek et al., 2007; Islam et al., 2012).

A WGA chitin assay (Ayliffe et al., 2013) was performed on root, leaf (all leaves) and crown tissue of *Fusarium* infected WT and *rdd* plants to determine where the majority of the fungal biomass is located. Fungal biomass increased over a 6 day period and was located predominantly in root and crown tissue, with only limited amounts of fungal growth occurring in leaf tissue (**Figure 1A**). As leaf vein clearing is a major symptom of *Fusarium* infection (Pietro et al., 2003; Michielse and Rep, 2009) leaf petioles (section ii) were excised and compared with the remaining leaf tissue (section iii) as well as shoot tissue (section iv) in a single experiment, however, no obvious difference in *Fusarium* accumulation was apparent between these two tissues (**Figure 1B**). Leaf vein clearing is therefore not a direct consequence of *Fusarium* accumulation in this tissue. Interestingly, in the *rdd* mutant increased fungal biomass was observed in crown tissue at 6 dpi when compared with the equivalent WT tissue.

### Expression of Defense-Related Genes Is Tissue-Specific, Highly Responsive to *Fusarium* Infection and Compromised in *rdd*

Having established the preferential growth patterns of *Fusarium* in Arabidopsis tissues shown above we investigated if defense-related genes containing promoter TE sequences are expressed in a tissue-specific manner upon infection. We selected six defense-related genes which we previously identified to be strongly downregulated in the *rdd* mutant compared to WT and which showed induction upon *Fusarium* infection (Le et al., 2014) (**Table 1**, genes 1–6). We analysed their expression in leaf (iii), midrib and petiole (ii), and the remaining shoot tissue (iv) (**Figure 1B**). These genes showed either similar levels of expression (At5G24210, At2G15040, At1G58602, and At4G09420) or displayed a distinct expression pattern among the three aerial tissue types (At5G39110, At5G38550) (Supplementary Figure S2). To simplify the analyses, all aerial tissues (shoot tissue) were combined and compared with root tissue in subsequent experiments. It is noteworthy that significant gene induction occurred in shoot tissues despite the reduced fungal colonization when compared with root tissue.

**Table 1:** List of all Arabidopsis genes analyzed in this study for their root/shoot expression and DNA promoter methylation levels.

#	Gene ID	Description
1	At1G58602	LRR and NB-ARC domain containing disease resistance protein
2	At5G39110	RmlC-like cupins superfamily protein
3	At4G09420	Disease resistance protein (TIR-NBS class)
4	At5G24210	Alpha/beta-Hydrolase superfamily protein
5	At5G38550	Mannose-binding lectin superfamily protein
6	At2G15040	pseudogene, disease resistance protein related
7	At4G33710	CAP (Cysteine-rich secretory proteins, Antigen 5, Pathogenesis-related 1 protein)
8	At4G33720	CAP (Cysteine-rich secretory proteins, Antigen 5, Pathogenesis-related 1 protein)
9	At4G11170	RMG1 (Resistance Methylated Gene 1), NB-LRR disease resistance protein

**FIGURE 2:**
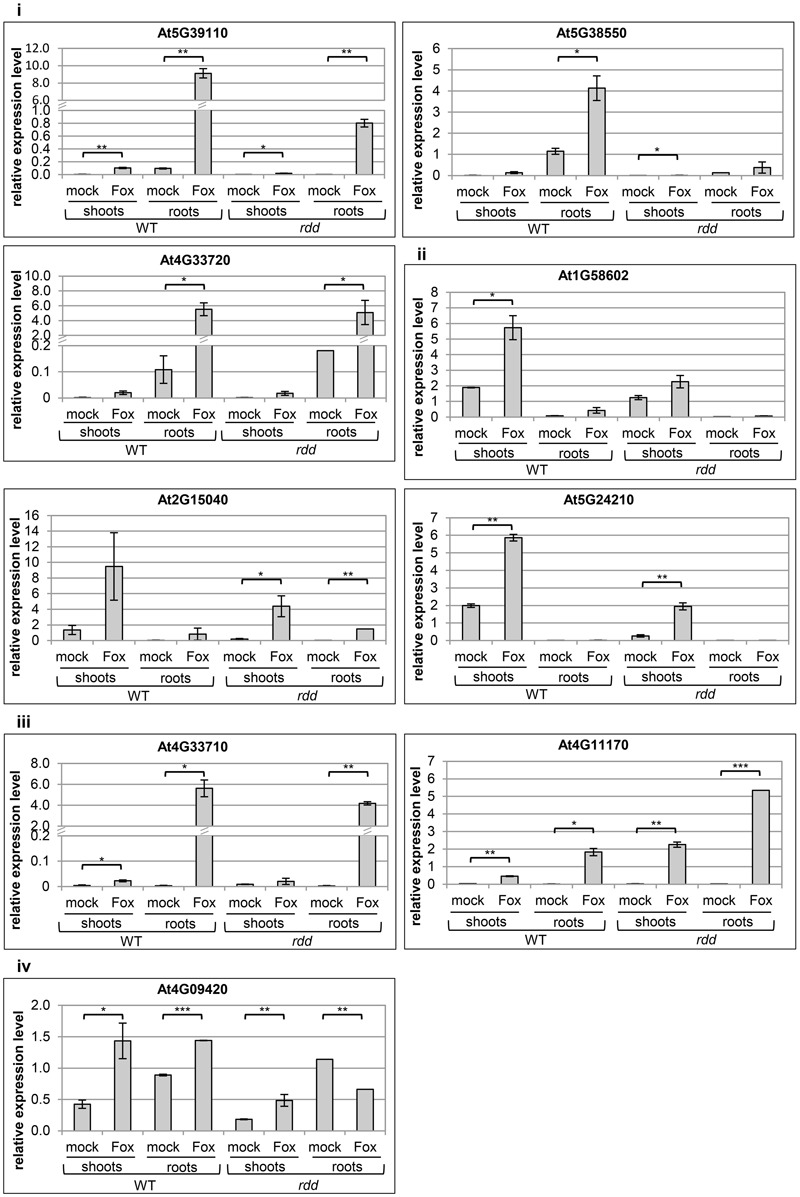
Expression levels of defense-related genes in WT and *rdd* root and shoot samples. Plants were infected with *Fusarium* (Fox) or mock treated and biological duplicate samples were harvested at 3 dpi. Roots and shoots of five individual plants per replicate were separated at the epicotyl/hypocotyl junction and RNA isolated for expression analysis. Root expression is induced for gene At5G24210 following *Fusarium* infection, but this induction is not significant and at such low levels that it was not feasible to portray this in the figure. Expression levels are relative to *actin2* and error bars indicate standard deviation. Significance levels used for the Student’s *t*-test analysis were: ^∗^*p* ≤ 0.05; ^∗∗^*p* ≤ 0.005; ^∗∗∗^*p* ≤ 0.0005; non-significant pairings are omitted to simplify the figure.

The expression profile of nine defense-related genes containing promoter TE sequences was analyzed in shoot and root tissues of WT and *rdd*, including all six *Fusarium*-inducible genes mentioned above and three additional defense-related genes (**Table 1**), the two neighboring genes AT4G33710 and AT4G33720 (both CAP superfamily proteins, PR1-related), and AT4G11170 (RMG1, NB-LRR disease resistance protein), which was shown to be induced by the bacterial elicitor *flg22* (Yu et al., 2013). As shown in **Figure 2**, these genes in general showed differential expression between shoots and roots, and displayed four types of *Fusarium*-responsive expression patterns; (i) expression detectable under mock conditions only in roots, but induced in both roots and shoots upon *Fusarium* infection (At5G39110, At5G38550, and At4G33720), (ii) expression detectable under mock conditions only in shoots, but induced in roots and shoots following *Fusarium* infection (At1G58602, At2G15040, and At5G24210), (iii) expression detectable in both shoots and roots but only following *Fusarium* infection (At4G11170, At4G33710), and (iv) expression detectable in both shoots and roots under mock conditions and induced by *Fusarium* infection in both tissues (At4G09420) (Supplementary Table S2). The level of induction was gene-specific with *Fusarium*-induced expression levels in the *rdd* mutant usually being lower than in WT plants. Consistent with previous reports using the *ros1* DNA demethylase single mutant (Yu et al., 2013), At4G11170 showed higher expression in the *rdd* mutant following infection. Furthermore, we observed tissue-specificity at the level of gene induction following infection for almost all genes (Supplementary Table S2).

These results highlight the advantage of analyzing specific tissues over whole plant samples given the tissue specificity of *Fusarium*-responsive defense-related gene expression coupled with the preferential pathogen accumulation in specific tissues. For example, using whole plant tissues we previously detected a 10-fold upregulation of gene At5G39110 upon *Fusarium* infection at 5 dpi (Le et al., 2014), whereas in the present study a 100-fold induction by *Fusarium* infection was detected when analyzing root tissue alone (**Figure 2**). Gene expression changes in specific tissue types are therefore not accurately determined when using whole plant tissue for analyses.

### Promoters of Defense-Related Genes Are Hypermethylated in the *rdd* Mutant

**FIGURE 3:**
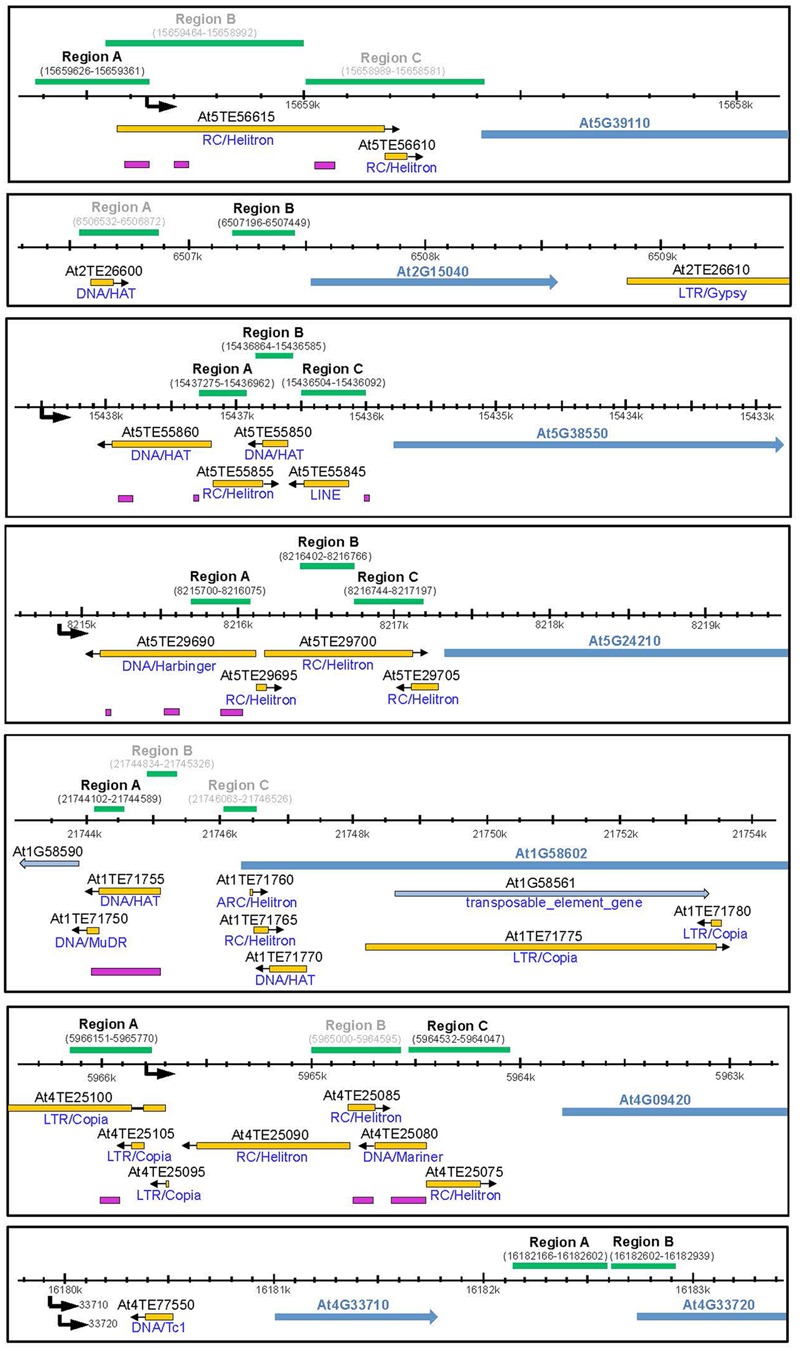
Schematic diagram showing promoter regions of defense-related genes subjected to bisulfite sequencing. Schematics were created according to TAIR10 with the gene model and name indicated by blue arrows. All genes of interest are displayed in 5′–3′ orientation. Neighboring genes models and TE genes are indicted by light blue arrows. Natural transposons, as annotated by TAIR, are shown by yellow bars with the orientation indicated by a black arrow and the TE family indicated below each bar. Regions targeted by small RNAs are indicated by pink bars (ASRP; http://asrp.danforthcenter.org/). Promoter start sites were predicted using the starPRO database (Baev et al., 2010) and are indicated by a black arrows. Genomic regions (coordinates in brackets) subjected to bisulfite sequencing are indicated above by green bars. Regions for which data could be obtained are indicated in black, regions that did not yield a PCR product are indicated in gray.

To investigate the molecular basis for the differential expression of these genes in *rdd* and WT plants the DNA methylation level of the promoter region of all the above genes, except At4G11170 and At4G33710, was examined (**Table 1**, genes 1–6 and 8). In particular, short TE sequences in promoter regions were examined as they are often targeted by RdDM (**Figure 3**, pink bars) and we have previously shown differential cytosine methylation of these sequences in *rdd* plants (Le et al., 2014). DNA was isolated from the same samples used for gene expression analysis and cytosine methylation analysis undertaken for genomic regions of interest (**Figure 3**, green bars). Methylation levels were determined by sequencing PCR amplified products derived from bisulfite treated DNA (for primer sequences see Supplementary Table S1).

**FIGURE 4:**
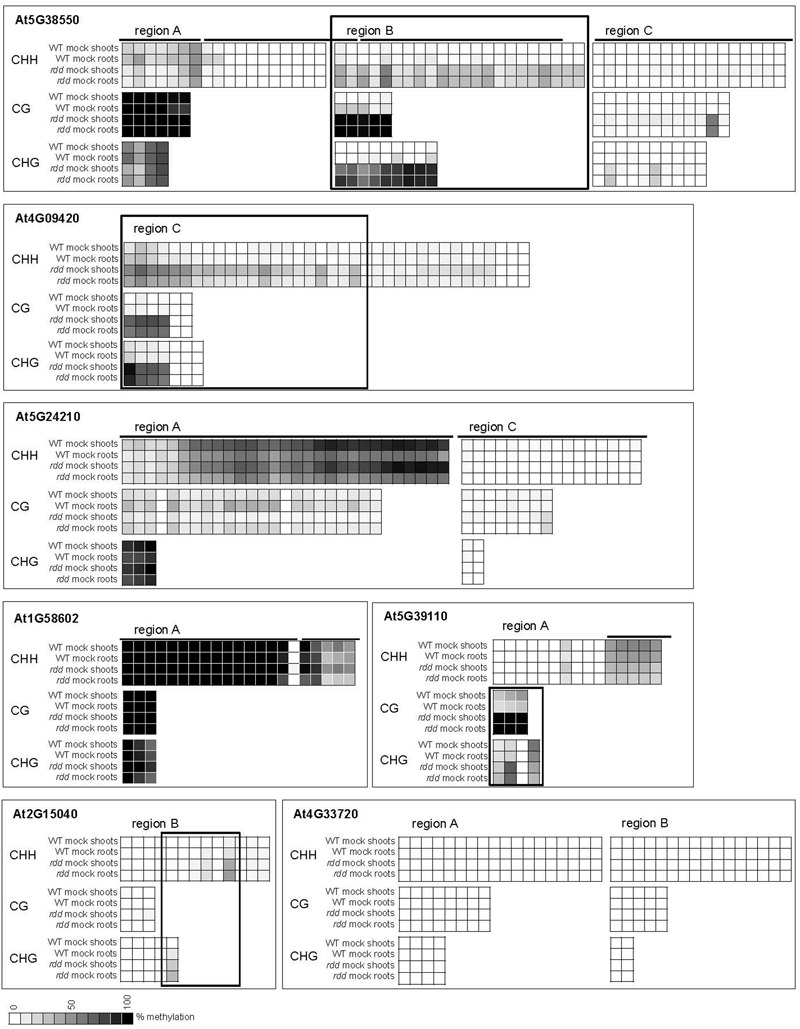
DNA methylation levels of defense-related gene promoters. Plants were infected with *Fusarium* or mock treated and samples harvested at 3 dpi. Methylation percentage was calculated using Mutation Surveyor and the mean methylation level for each site is represented as a heatmap. Each square represents a single cytosine and lines above graphs show location of TE elements. Boxes indicate regions where the majority of cytosines showed significant differential methylation (*p* ≤ 0.05) between *rdd* and WT. The TSS for each gene is located to the right of the sequences shown here (see **Figure 3**). Extended regions of non-differential methylation have been removed for easier visualization. *Fusarium* infection data were largely similar and are not shown for easier visualization. For the complete dataset including Student’s *t*-test *p*-values see Extended Data File 1.

Increased promoter methylation levels in regions near (∼500–1000 bp) the putative gene transcription start sites (TSSs) were consistently observed in the *rdd* mutant when compared with WT for most genes analyzed. Increased DNA methylation in the mutant was largely irrespective of tissue type or treatment with only minor localized differences occurring between different tissues and infected/uninfected tissues (for full dataset see Extended Data File 1). Hypermethylation in *rdd* was particularly obvious for genes At5G38550, At4G09420 and At5G39110, and to a lesser extent for gene At2G15040 (**Figure 4**, black boxes). Specifically for region B of gene At5G38550 and region C of gene At4G09420, cytosine methylation levels in all three sequence contexts were low in the WT and comparatively higher in *rdd*. Increased CG methylation was also observed in region A of the At5G39110 gene in *rdd* plants. Similarly, some CHH and CHG sites in region B of gene At2G15040 also showed hypermethylation in *rdd*. In contrast, for two regions of gene At5G24210 (region A – highly methylated, region C – lowly methylated) and the highly methylated region A of gene At1G58602, no clear differences in methylation pattern were observed between mutant and WT plants (**Figure 4**). However, analysis of whole genome bisulfite sequencing data (Stroud et al., 2013) showed hypermethylation in *rdd* plants of region B of gene At5G24210 (between the two regions analyzed here) and region B of gene At1G58602 (downstream of region analyzed here) (Supplementary Figure S3). No DNA methylation was detected for gene At4G33720 (**Figure 4**).

**FIGURE 5:**
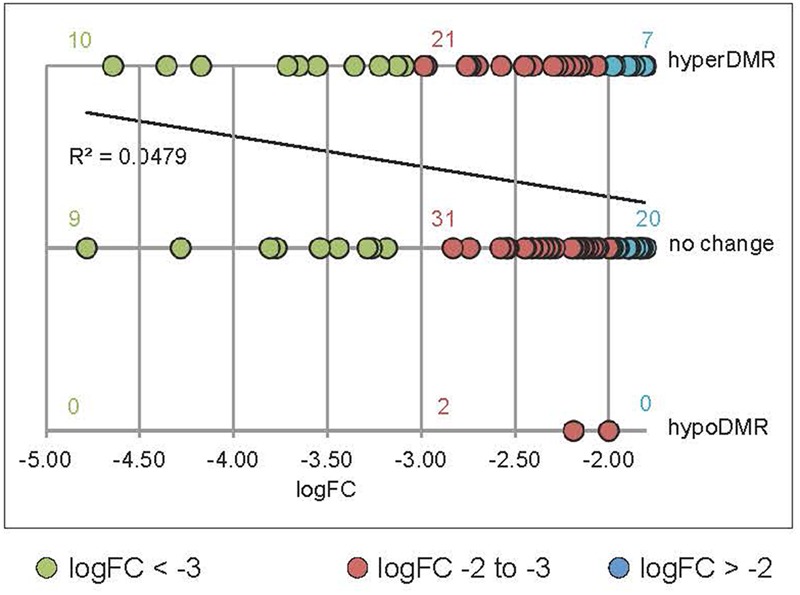
Positive correlation between the level of downregulation in *rdd* and hypermethylation within 1 kb of the genes TSS’s. Data were obtained by interrogating a publicly available database (Stroud et al., 2013) for *rdd* and WT DNA methylation levels. *rdd*-downregulated protein coding genes (Le et al., 2014) were analyzed (downregulation logFC between –4.78 and –1.8). Transposable element (TE) genes were eliminated from this analysis. For multiple splice variants, only the most downregulated variant was included. The number of gene promoters with hyper-, hypomethylation (hyperDMR, hypoDMR) and no change in *rdd* compared to WT were plotted against the level of downregulation (logFC). Linear regression of all data shows a positive correlation between the level of downregulation and hyperDMR.

We undertook further analysis of promoter methylation for over 100 of the most significantly *rdd*-downregulated genes using the Stroud et al. (2013) bisulfite sequencing data which revealed a positive, quantitative correlation between gene repression and the presence of hypermethylation within 1kb of gene TSS’s. Ten (53%) out of 19 highly downregulated *rdd* genes (logFC < -3.0; Le et al., 2014) had hypermethylated promoter regions close to gene TSS’s, compared with 39% (21/54 genes, logFC -3.0 to -2.0) and 26% (7/27, logFC > -2.0) of genes that were less severely repressed in the *rdd* background (**Figure 5**). Together these results suggest that DNA demethylases specifically target TE sequences in promoter regions of defense-related genes for active demethylation and this demethylation process is essential to maintain gene expression levels.

### Root and Shoot Tissues Show Low-Level Differential Methylation Patterns of Defense-Related Gene Promoters

**FIGURE 6:**
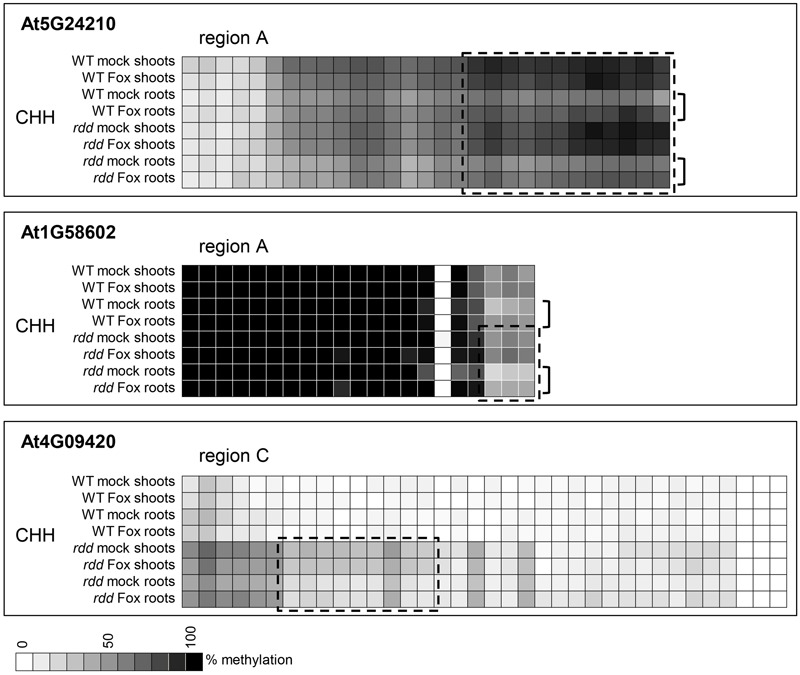
Tissue specific differences in CHH methylation in defense-related gene promoters occur in a distinct localized manner. Data are identical to **Figure 4** but selected regions of CHH methylation are shown for all datasets. The order in which the data are represented varies to **Figure 4** to highlight the differences. Boxes highlight regions where the majority of cytosines show significant hypermethylation (*p* ≤ 0.05) in shoots compared to roots. Brackets highlight regions where *Fusarium* infection increased CHH methylation in roots compared to shoots (*p* ≤ 0.1). Student’s *t*-test *p*-values can be found in the Extended Data File 1.

While WT and *rdd* plants show clear differential methylation in promoter regions, only low levels (10–15% change) of localized methylation differences were observed for the equivalent sequences in root and shoot tissues of the same genotype (Supplementary Table S3). Methylation differences between these tissues were most apparent in the CHH context in gene At5G24210 where both *rdd* and WT plants showed increased CHH methylation in shoots compared with roots, while in genes At4G09420 and At1G58602 this increased shoot CHH methylation was only observed in the *rdd* genotype (**Figure 6**, boxes). Differential methylation was particularly localized for genes At1G58602 and At4G09420, affecting only few cytosine residues. *Fusarium* infection increased CHH methylation in gene At5G24210 and to a lesser extent in At1G58602 in WT and *rdd* roots, but not shoots (**Figure 6**, brackets).

Although promoter methylation is generally considered to repress gene expression, four of the genes analyzed showed a positive correlation between hypermethylation and increased expression in a specific tissue (Supplementary Table S3). The context of this hypermethylation was primarily CHH and not CG, which is consistent with a previous observation that repressed gene expression in *rdd* plants tended to be associated with CG hypermethylation but not CHH hypomethylation (Le et al., 2014). Interestingly, three of these four genes showed an increase in CHH methylation in response to *Fusarium* infection in tissues in which expression is induced upon infection. These results suggest that CHH methylation, indicative of RdDM, may play a regulatory role in the expression of defense-related genes during stress response, as previously speculated (Le et al., 2014).

### ROS1 Is Essential for the Regulation of *rdd*-Downregulated Defense-Related Genes

*ros1, dml2*, and *dml3* were previously shown to have some functional redundancy in the response of Arabidopsis to *Fusarium* infection (Le et al., 2014). To dissect the contribution of each demethylase gene to DNA methylation and gene expression changes in the *rdd* mutant and in response to *Fusarium* infection, single demethylase mutants (Penterman et al., 2007) were analyzed. The *rdd* triple mutant showed the strongest symptoms to *Fusarium* infection amongst all the genotypes assayed, confirming that some functional redundancy exists amongst the three demethylases (Supplementary Figure S4). *dml3-1* showed a similar phenotype to WT Col-0, whereas *dml2-1* showed more leaf yellowing than WT plants, with *ros1-3* displaying the strongest disease symptoms amongst the single mutants. This suggests that ROS1 is the dominant DNA demethylase in the regulation of *rdd*-controlled defense-related genes. Although the *rdd* mutant used in our analysis carried different mutations to the single mutants analyzed here (for details see Materials and Methods), the equivalent *rdd* triple mutant comprising these three single mutants showed comparable symptoms to the *rdd* mutant analyzed here.

**FIGURE 7:**
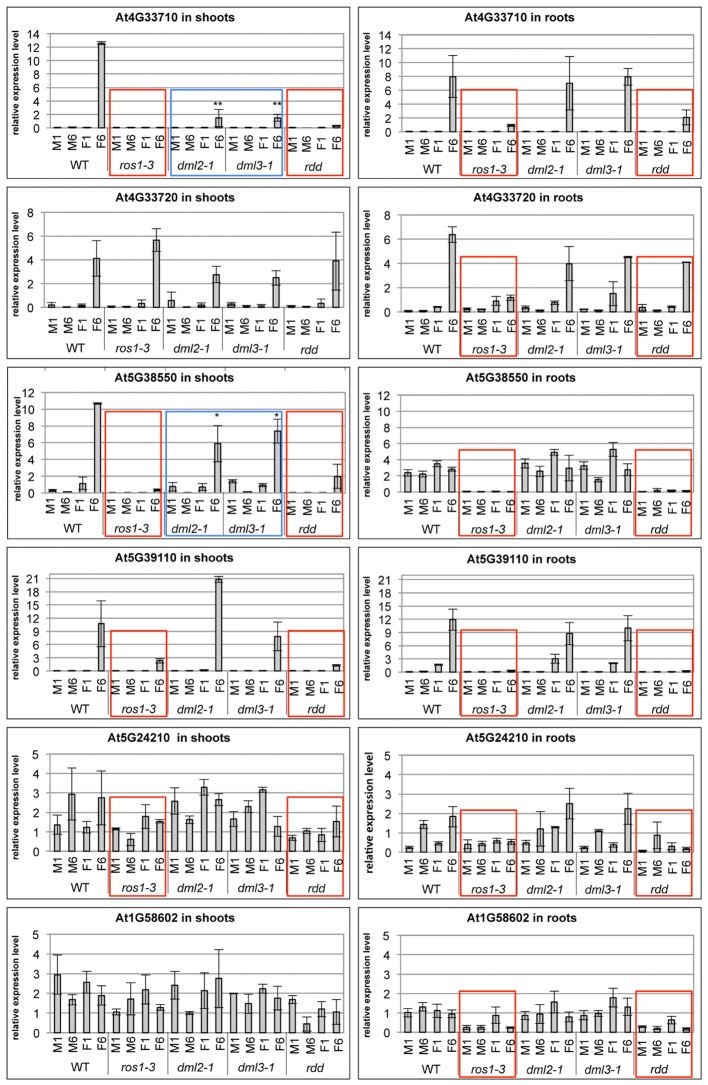
The *ros1-3* mutant shows a similar gene expression profile of defense-related genes as the *rdd* mutant. Plants were *Fusarium* infected (F) or mock treated (M) and the expression level in shoots (left panels) and roots (right panels) examined at 1 and 6 dpi. Expression levels of defense-related genes are shown relative to *FDH*; comparable results were obtained using *actin2* as reference. Bars show the average of three independent biological replicates with error bars indicating standard deviation. Expression profiles that are similar between *rdd* and *ros1-3* are indicated with red boxes, while expression profiles intermediate between WT and *rdd* in the *dml2-1* and *dml3-1* mutants are indicated by blue boxes. ^∗^*p* ≤ 0.1 and ^∗∗^*p* ≤ 0.05, according to Student’s *t*-test.

Defense-related gene expression and promoter DNA methylation levels were also analyzed in root and shoot tissue of single mutants and compared with WT and *rdd* plants. Suppressed gene expression under mock conditions and reduced gene induction upon *Fusarium* infection, characteristic of the *rdd* mutant, was largely replicated in the *ros1-3* mutant (**Figure 7**, red boxes). In contrast, *dml2-1* and *dml3-1* mutants showed expression profiles similar to WT. Repression of basal and pathogen-induced gene expression in *ros1-3* occurred in both root and shoot tissue for At3G33710, At5G38550, At5G39110, and At5G24210 while suppression of At3G33720 and At1G58602 occurred in root tissue only (**Figure 7**, red boxes). These results further indicated that ROS1 is the dominant DNA demethylase required for positive regulation of these defense-related genes.

However, *ros1-3* does not entirely phenocopy *rdd*, as downregulation of basal expression of At5G24210 (both shoots and roots) and At1G58602 (shoots only) was less compromised in this mutant compared to *rdd*. In addition, At4G33710 and At5G38550 showed reduced levels of *Fusarium*-induced gene induction in shoots of *dml2-1* and *dml3-1* compared to WT (**Figure 7**, blue boxes). These data suggest that DML2 and DML3 also contribute to the *rdd-*mediated downregulation of some genes.

**FIGURE 8:**
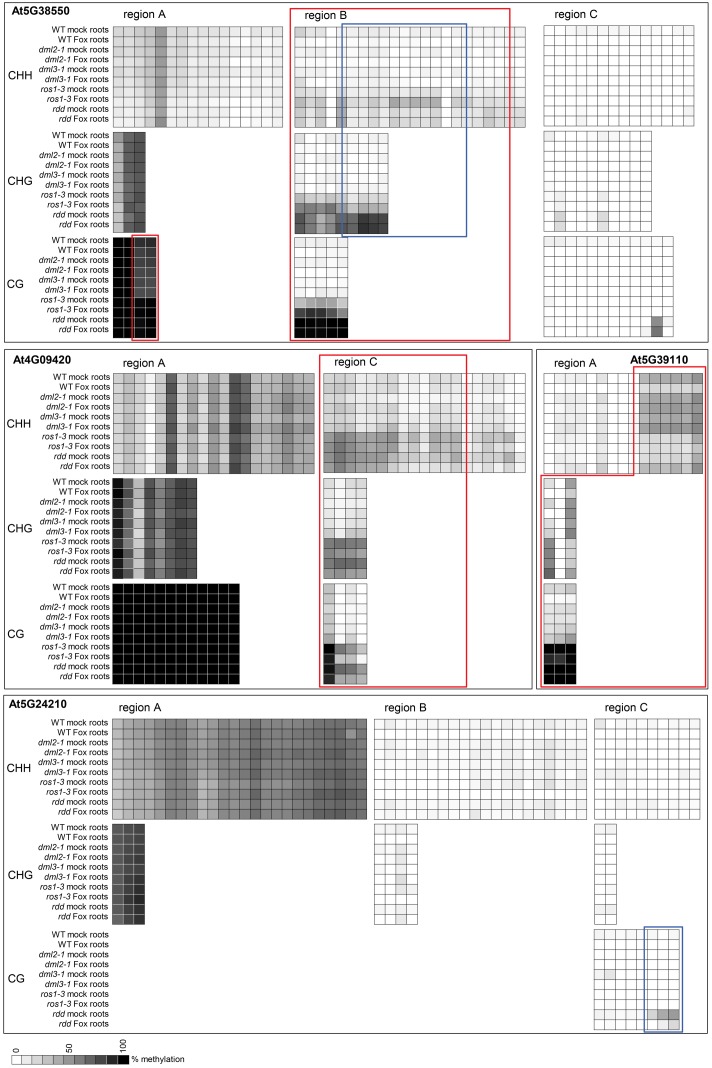
The *ros1-3* mutant shows hypermethylation of TE sequences in defense-related gene promoters, similar to *rdd*. DNA methylation in single demethylase mutants *ros1-3, dml2-1, dml3-2* as well as the triple mutant *rdd* and WT was analyzed at 3 dpi. Methylation level was determined using Mutant Surveyor and the average of two biological replicates is shown as a heatmap. Each square represents a single cytosine in the sequenced region. Cytosines with significant hypermethylation (*p* ≤ 0.1) in *ros1-3* (and *rdd*) compared to WT are indicated by red boxes. Regions where *rdd* and *ros1-3* show significant differential methylation (*p* ≤ 0.1) are highlighted by blue boxes. Shoot data were largely similar and are not shown for easier visualization. Extended regions of non-differential methylation have been removed for easier visualization. The full dataset including the *t*-test *p*-values can be found in Extended Data File 2.

To verify that the repressed gene expression in *ros1-3* and to a lesser extent *dml2-1* and *dml3-1* is due to localized DNA hypermethylation in promoter regions of these genes, bisulfite sequencing was undertaken on four genes (At4G09420, At5G38550, At5G39110, and At5G24210) in both root and shoot tissue (for full dataset see Extended Data File 2). Regions that showed increased methylation in the *rdd* mutant also showed significant hypermethylation in *ros1-3* (**Figure 8**, red boxes). This increased methylation was not observed in *dml2-1* and *dml3-1*, which had similar methylation levels to WT Col-0. At5G24210 was an exception and showed no clear difference in methylation levels among the five genotypes (WT, *rdd, ros1-3, dml2-1*, and *dml3-1*) except in CG methylation in region C (**Figure 8**, blue box).

Interestingly, for gene At5G38550, the level of methylation in region B was lower in *ros1-3* than in *rdd*. Similarly, the level of CG methylation in region C of At5G24210 was also lower in *ros1-3* than in *rdd* (**Figure 8**, blue boxes). These results suggest that while ROS1 is the dominant player among the three DNA demethylases, it does not account for all DNA demethylase activities, consistent with the existence of functional redundancy as suggested by the disease phenotypes. The methylation data again supports a direct role for localized differential DNA methylation resulting in repressed gene expression in *ros1-3* and *rdd*. The contribution of DML2 and DML3 to methylation could not be confirmed by bisulfite sequencing as only subtle differences were observed between these two mutants and WT Col-0 for the four genes analyzed.

Similar to the observation with WT and *rdd* (**Figure 6**, brackets), localized differences in methylation were also observed between roots and shoots and between *Fusarium*-infected and mock-treated *ros1-3* plant tissues (Supplementary Figure S5). However, these methylation differences were not striking and unlikely to account for the tissue-specific or *Fusarium*-responsive expression patterns of the respective genes in *ros1-3*.

## Discussion

### DNA Demethylase-Regulated *Fusarium*-Response Genes Show Tissue-Specific Expression Patterns

In our previous study whole plant tissue of WT Col-0 and the *rdd* mutant was used to analyze gene expression and DNA methylation differences between each genotype and between *F. oxysporum*-infected and mock-treated plants (Le et al., 2014). We observed that the repressed expression of a subset of defense-related genes in *rdd* was associated with altered DNA methylation of short TE sequences in the gene promoters, which suggests that these genes are regulated by DNA demethylases at these TE sequences. We also observed that these *rdd*-downregulated genes tend to show increased expression upon *Fusarium* infection, however, this increased expression is not associated with a clear change in DNA methylation of these short TE sequences. This suggested that *Fusarium*-responsive gene expression might be tissue-specific and changes in DNA methylation might occur in a tissue-specific manner and escape detection in whole plant tissue analysis.

In this study we first examined the spatiotemporal distribution of *F. oxysporum* in Col-0 and *rdd* plants. The majority of fungal biomass was restricted to root tissue at 1–3 dpi and remained largely confined to the roots even at 6 dpi. In the aerial part of the plants, fungal biomass was detected at a significant level only in the crown tissue at the later stage of infection. Very little fungal growth was observed in tissue surrounding leaf veins or expanded leaves, even after leaf yellowing symptoms were visible. In addition, the increased fungal disease symptoms in *rdd* were not associated with a large increase in fungal biomass. These results suggest that the increased symptoms in *rdd* leaf tissues are not caused by direct fungal colonization, but rather are a secondary response to infection.

The differential accumulation of fungal biomass between shoot and root tissues prompted us to investigate the expression pattern of the *rdd*-downregulated, *Fusarium*-responsive, defense-related genes in these two tissue types in both *rdd* and WT Col-0 plants. Tissue-specific or tissue-preferential expression was observed for eight of the nine genes analyzed, while all nine genes were induced by *Fusarium* infection. Interestingly, only At5G38550 was identified to be induced in roots by *Fusarium* in a previous transcriptome-wide study (Lyons et al., 2015). These results demonstrate that some defense-related genes are highly regulated in a tissue-specific manner, thereby highlighting the necessity for separating tissues in gene expression studies.

### ROS1 Is the Major Demethylase Mediating Active Demethylation of Promoter TE Sequences

The majority of *rdd*-downregulated defense-related genes analyzed accumulated increased DNA methylation in TE sequences located in promoter regions close to gene TSS in the *rdd* mutant. Expression of these defense-related genes in *rdd* were still inducible by *Fusarium* infection, but the level of induced expression did not reach that of WT plants, consistent with the increased DNA methylation of promoter TE sequences repressing gene expression under both mock and *Fusarium* infection conditions. These observations indicate that DNA demethylases positively regulate defense-related gene expression under both these conditions.

To understand the role of each individual DNA demethylase present in the *rdd* triple mutant, we analyzed single demethylase mutant lines *ros1-3, dml2-1* and *dml3-1* for *Fusarium* susceptibility, defense-related gene expression and promoter methylation. *Dml3-1* plants showed a similar response to *Fusarium* infection as WT, while *dml2-1* plants were only slightly more susceptible than WT plants. However, *ros1-3* plants were highly susceptible although the symptoms were less severe than those observed for *rdd*. These data suggest that ROS1 is the dominant DNA demethylase that regulates genes involved in *Fusarium* resistance in Arabidopsis. Consistent with this hypothesis, gene expression analysis showed that expression patterns of all six defense-related genes analyzed in *ros1-3* was similar to the expression profiles observed in *rdd*. Interestingly, both *dml2-1* and *dml3-1* mutants did not always show a WT-like gene expression profile; for three of the six genes the expression level in these two mutants was intermediate between WT and *rdd*/*ros1-3*. This suggests that DML2 and DML3 do contribute to the regulation of some genes, possibly in a tissue-specific manner.

Similar to *Fusarium* infection and gene expression analysis, DNA methylation analysis showed that loss of ROS1 results in hypermethylation of promoter TE sequences, whereas DNA methylation profiles of *dml2-1* and *dml3-1* were virtually identical to those of the WT Col-0 plants. This further confirms that ROS1 is the dominant demethylase required to positively regulate these defense-related genes by actively demethylating promoter TE sequences. However, for one gene (At5G38550, region B) the level of methylation in *ros1-3* was not as high as in *rdd*, and some regions showed tissue-specific differential methylation in *ros1-3*. These results further suggest that DML2 and DML3 participate in the demethylation of some genes, and that they may play a role in fine-tuning methylation or in tissue-specific methylation. This notion is also supported by the observation that some genes show intermediate expression patterns in shoot tissue in both *dml2-1* and *dml3-1*.

### Tissue-Specific and *Fusarium*-Inducible Gene Expression Patterns Are Not Directly Regulated by Differential DNA Methylation

Unlike the clear differential DNA methylation occurring between *rdd*/*ros1-3* and Col-0, we only observed small, localized methylation differences in promoter TE sequences when root and shoot tissues were compared within a genotype, despite distinct expression patterns existing between these tissues. These differences in methylation level occurred mainly in the CHH context, particularly in the *rdd* mutant, and usually involved a few cytosine residues in close proximity. This hyper-CHH methylation in a specific tissue type tended to be associated with increased gene expression. For instance, shoots showed higher levels of CHH methylation than the roots for genes At5G24210 and At4G09420 and these two genes were expressed specifically in shoots. However, the lack of widespread methylation differences between roots and shoots suggests that tissue-specific expression patterns are not determined by DNA methylation in promoter TE regions. Our observation is consistent with a previous report that showed no significant global methylation differences between roots and shoots in the Arabidopsis Col-0 ecotype (Widman et al., 2014). This study identified 2424 genes as differentially expressed between the two tissues. Although these genes showed some enrichment of differential methylation between tissues, only one in every 173 cytosines was actually differentially methylated (Widman et al., 2014).

Similar to the small methylation difference between root and shoot tissues, no widespread methylation changes were observed between *Fusarium*-infected and mock-treated plants in promoter TE sequences of the defense-related genes analyzed. Only one gene (At5G58602) was found to have increased CHH methylation in response to *Fusarium* in both tissues in *rdd* plants. These data suggest that the *Fusarium*-inducible expression patterns of these genes are independent of DNA methylation in the promoter TE sequences.

### Possible Mechanisms for the Regulation of DNA Methylation-Controlled Defense-Related Genes

Our results show that the repressed expression of defense-related genes in *rdd* and *ros1-3* plants is largely explained by differential DNA methylation of short promoter TE sequences repressing gene expression. However, spatiotemporal regulation of these same genes in shoot and root tissues, and upon *Fusarium* infection, appears independent of DNA methylation. TE insertions in the mammalian genome are suggested to contribute *cis*-acting elements to adjacent genes, thereby conferring altered gene regulation (Bourque et al., 2008; Makarevitch et al., 2015). Similarly, in maize stress-response genes are enriched for TE insertions near genes and these TE elements may provide enhancers for stress-induced gene expression (Makarevitch et al., 2015). However, a genome-wide methylation analysis of maize plants after abiotic stresses indicated that stress-responsive gene expression was not usually associated with DNA methylation changes (Eichten and Springer, 2015).

In light of these studies and our own results, it is conceivable that the *Fusarium*-responsive and tissue-specific expression patterns of these defense-related genes could be due to *cis*-elements encoded by adjacent TEs. This is consistent with TEs being inducible by environmental stresses and therefore containing stress-responsive elements (Capy et al., 2000; Casacuberta and Gonzalez, 2013). In support of this hypothesis, we searched the upstream region (250–1500 bp) of *rdd*-downregulated genes for transcription factor (TF)-binding site sequence using the AthaMAP database (Steffens et al., 2004). Enrichment for bZIP_DOF and MYB class TF-binding sites was observed, particularly in the differentially methylated TE regions present within these gene promoters (Supplementary Table S4). For instance, 38 of the most downregulated protein-coding genes in the *rdd* mutant (Le et al., 2014) had bZIP_DOF and MYB-type TF-binding sites in 45–50% of the promoters, which was twice the frequency when compared with a randomly selected set of genes (Supplementary Table S4). In addition, some of these detected putative *cis*-elements have known roles in stress-inducible or tissue-specific gene expression (Villain et al., 1996; Zhou, 1999; Yanagisawa, 2004; Luo et al., 2010; Noguero et al., 2013; Behringer and Schwechheimer, 2015; Gupta et al., 2015) (Supplementary Table S5).

However, TEs are the main target of RdDM, and consequently, while these TE sequences are required to confer tissue-specific or pathogen-inducible expression patterns, they are also subject to DNA methylation affecting the transcriptional activity of the adjacent genes. This is consistent with DNA hypermethylation of these regions in *rdd* repressing gene expression (Le et al., 2014; and this study). DNA demethylases are therefore required to minimize the DNA methylation level, thus allowing accessibility of *Fusarium*-inducible TFs and concomitant induced gene expression. Further genome-wide investigations are required to substantiate this model, but the combination of TE insertions, RdDM and DNA demethylases may play a major role in the evolution of plant transcriptional responses to phytopathogens.

## Author Contributions

Conceived and designed the study: US, M-BW, MA, KK; performed experiments: US, JL; analyzed the data: US, JL; provided reagents: MA; wrote the manuscript: US, M-BW, KK, MA.

## Conflict of Interest Statement

The authors declare that the research was conducted in the absence of any commercial or financial relationships that could be construed as a potential conflict of interest.
