# Supplementary material for: DNA-Demethylase Regulated Genes Show Methylation-Independent Spatiotemporal Expression Patterns
Source: Front Plant Sci. 2017 Aug 28;8:1449. doi: 10.3389/fpls.2017.01449 (PMC5581395; doi:10.3389/fpls.2017.01449)
Supplement: Supplementary file 1 [file Table_1.PDF]

Table S1: Primers used in this study. Forward, reverse and nested primers are indicted with F, R, N respectively. Bisulfite PCR was carried out using F and R for primary PCR and then a subsequent PCR with a nested primer. Y = C+T, R = A+G

gene ID	description	qPCR primer sequence	
At4G09420	Disease resistance protein (TIR-NBS class)	F	TCAAGAGGGCACTCAAACG
		R	GCTTCCAGGAAAACACAAGC
At1G58602	LRR and NB-ARC domain containing disease resistance protein	F	TGGAAGAAGAGATTGTCGGAAGGAGG
		R	TCCTGGCTCTGTCTGTGATTGCTG
At5G39110	RmlC-like cupins superfamily protein	F	CTTATCGGCTTTGGTCATTTCC
		R	AACACCATTTTTGAGGTCGCC
At5G38550	mannose-binding lectin	F	GCATATTATCGCCCGATTCC
		R	CAGATGAAAGGGTCTTGTTGC
At2G15040	pseudogene, disease resistance protein related	F	AACAAAGAGACGCAATTCTCG
		R	GAGGCAGCTGGAGCTAAGG
At5G24210	Alpha/beta-Hydrolase superfamily protein	F	ACGCAAAGGAAAGAGACAGG
		R	CCAATCACTCACATCACTTGG
At4G33710	CAP (Cysteine-rich secretory proteins, Antigen 5, Pathogenesis-related 1 protein)	F	TCTCATTCACTCAAACTCACGC
		R	CCACATTGTTTTCCAGCACGG
At4G33720	CAP (Cysteine-rich secretory proteins, Antigen 5, Pathogenesis-related 1 protein)	F1	ATGGAGAGAACATCGCTTGG
		R1	CCGAGTTTCTCCACACAACC
		F2	CCCGTAACTATGCTAACCAGC
		R2	GCACATGTATTGGAATCATAATCG
At4G11170	RMG1 (Resistance Methylated Gene 1), NB-LRR disease resistance protein	F	CCAAAGCATTTGGATTCACC
		R	GGCTCCAGGAACTGTAAACC
At3G18780	Actin2	F	AGTGGTCGTACAACCGGTATTGT
		R	GATGGCATGAGGAAGAGAGAAA
At5G43940	FDH	F	TTGGACTTGCTGTTGCCGA
		R	AAGTTCCCCATCCCTTGTGAC

gene ID	region	Bisulfite PCR primer sequence
At4G09420	CF	AATAAATATTTATYTAATTAGTAAGGG
	CFN	GGAAATATAAAAYTTYTATYTTTGTGGG
	CR	CCTAATTTTRATTAAAACATTRCCTTC
At1G58602	AF	TTATTAGTYAGGGGYAAAATGG
	AFN	TTTYAYTAAAAGAATTAGAAAAGTG
	AR	AAAACRCAATTCAACATTTTCTC
At5G39110	AFN	GTTYTYTGATTTGGAGGAAATATGG
	AR	CARCCATTRTAAATAARTTCTAACTCC
	AF	GAAAYTSYGAGTTYTYTGATTTGGAGG
At5G38550	AF	GGTTAATAGTGAGTTTTTGTGGG
	AFN	TTGATTTTGAAYTTGTYGGGTTTTAG
	AR	ATAAAACTCAAACATATCTTTTCTC
	BF	AAAATAYTAAGATAGTAGGATAGTGA
	BR	CTRCAACTCTRRTTTRCAACCAAACC
	BRN	AAAAAATCTRCTCRTCCAATCCTAC
	CF	GGTTGAAYYYATTTGAAGATATATATAG
	CR	ATATRACAAACCTACTCTRTAAAACTT
	CRN	TTARAAACATAAAACCAACTTCAAC
At2G15040	BF	GGATAGTTAAAYYATATGATGGTTAG
	BFN	TGTGTAGGAGAYTAGGAATTGTG
	BR	ACATRRTTTCATTCTCATATATATCAC
At5G24210	AF	TGGYGAGAAAATATAATTTAGTGG
	AFN	TAGGTGGAATAGYGGTTTTTGG
	AR	TTTCTTCATCCAAATRATCCATCC
	CF	ATTTTGGATAAGGATAAGAGAAAG
	CFN	TGGGTTATYTAYGAYYGGGGGA
	CR	CCTTATATRTCAARTARTCAACAACTC
At4G33720	AF	GTTTAAAAYAAAATTATTYGTGGTGG
	AFN	GGGAAAAYAYAATAGTYAGYGATGTG
	AR	AAATRRCCAATTRATTAARTRTAATTATTTC
	BF	GTYAATTAYTATGGYGTGTAGYTATG
	BRN	CTTAARRRTCCCACCCCAACCTC
	BR	CARCCACCTTCTCRTCCCATC
psaA	F	CACCATTTARCTATTRCAATTCTTT
	R	ATGAYGTTGTTAGGATYTYATATAGG
