# Supplementary material for: DNA-Demethylase Regulated Genes Show Methylation-Independent Spatiotemporal Expression Patterns
Source: Front Plant Sci. 2017 Aug 28;8:1449. doi: 10.3389/fpls.2017.01449 (PMC5581395; doi:10.3389/fpls.2017.01449)
Supplement: Supplementary file 2 [file Table_2.pdf]

**Table S2: Summary of expression levels at 3 dpi in root and shoot tissues and changes in response to *Fusarium* based on RT-qPCR results obtained for WT (Figure 2). Basal expression levels were lower in *rdd*, but induction in response to Fusarium was similar to induction level observed in WT plants.**

| | Shoot tissue | | Root tissue | |
|---|---|---|---|---|
| **Gene ID** | **expression** | **Response to Fox** | **expression** | **Response to Fox** |
| At4G09420 | + | ↑↑ | ++ | ↑ |
| At1G58602 | +++ | ↑↑ | + | ↑ |
| At5G39110 | - | ↑ | + | ↑↑↑↑ |
| At5G38550 | - | ↑ | ++ | ↑↑↑ |
| At2G15040 | ++ | ↑↑↑↑ | - | ↑ |
| At5G24210 | +++ | ↑↑ | - | → |
| At4G33710 | - | → | - | ↑↑↑↑ |
| At4G33720 | - | ↑ | + | ↑↑↑↑ |
| At4G11170 | - | ↑ | - | ↑↑ |

| | | | |
|---|---|---|---|
| - | not expressed | ↑ | < 2fold |
| + | < 0.5 | ↑↑ | 2-3 fold |
| ++ | 0.5 – 1 | ↑↑↑ | 3-4 fold |
| +++ | > 1 | ↑↑↑↑ | > 4fold |
