# Supplementary material for: DNA-Demethylase Regulated Genes Show Methylation-Independent Spatiotemporal Expression Patterns
Source: Front Plant Sci. 2017 Aug 28;8:1449. doi: 10.3389/fpls.2017.01449 (PMC5581395; doi:10.3389/fpls.2017.01449)
Supplement: Supplementary file 3 [file Table_3.pdf]

Table S3: Tissue specific methylation differences in promoter regions of defense-related genes. Observed differences are small, between 10-20%. All comparisons are initially made between tissues only and then the effect of infection was considered. E.g. hypermethylation in roots means increased methylation in roots compared to shoots in uninfected tissue. Unless otherwise stated, infected samples showed the same methylation level as uninfected samples. Any observed difference have been listed, e.g. if only WT is mentioned, than the change has not been observed in *rdd*, but if neither is stated, then the difference was observed in both genotypes. If no remark has been made about the presence of differential methylation, then no clear difference was observed. For three genes the differential methylation is shown in

Gene	expression (Figure 2)	Region A	Region B	Region C
At5G38550	- root specific<br>- induced in both tissues	- hyper CHG in roots<br>- hyper CHH in WT roots<br>- CHH in WT roots increased by infection<br>- CHG in WT roots increased by infection<br>- CHG in WT shoots decreased by infection	- hyper CHG in *rdd* roots<br>- CHG in *rdd* roots increased by infection<br>- CHH in *rdd* roots increased by infection	no methylation
At5G24210	- shoot specific<br>- induced in shoots	- hyper CHH in shoots<br>- hyper CHG in shoots<br>- CHH in roots increased by infection	n.d.	no methylation
At4G09420	- expressed and induced in both tissues, slightly stronger in shoots	n.d.	n.d.	- hyper CHH in *rdd* shoots
At5G39110	- root specific<br>- induced dramatically in roots<br>- some induction in shoots	- hyper CG in WT shoots<br>- CHH in roots increased by infection<br>- CHH in WT shoots decreased by infection	n.d.	n.d.
At1G58602	- shoot specific<br>- induced in both tissues	- hyper CHH in shoots<br>- CHH increased in WT roots by infection<br>- CHH in *rdd* increased by infection<br>- CHG in *rdd* roots and WT shoot increased by infection<br>- CHG in *rdd* shoots decreased by infection	n.d.	n.d.
At2G15040	- shoot specific, low level<br>- induced in both tissues	n.d.	no methylation	n.d.
At4G33720	- root specific<br>- dramatically induced in roots<br>- some induction in shoots	no methylation	no methylation	n.d.
