# Supplementary material for: DNA-Demethylase Regulated Genes Show Methylation-Independent Spatiotemporal Expression Patterns
Source: Front Plant Sci. 2017 Aug 28;8:1449. doi: 10.3389/fpls.2017.01449 (PMC5581395; doi:10.3389/fpls.2017.01449)
Supplement: Supplementary file 4 [file Table_4.pdf]

Table S4: Analysis of the most prevalent TF binding sites within 250 bp to 1500 bp of the TSS of the 38 most downregulated protein-coding genes in the *rdd* mutant compared to a randomly selected gene list. Data were obtained from the AthaMAP database at stringency of 80%. Number represent total number of TF binding sites identified in all promoters analysed, percent shows the percentage of promoters that carry these sites, and distance to TSS was calculated as median distance. Grey highlighted are TF binding sites that seem enriched in defense-related gene promoters compared to the random gene list.

	most down-regulated genes (n=38)			random list of genes (n=38)		
**TF type**	**number**	**percent**	**distance**	**number**	**percent**	**distance**
bZIP_DOF	32	45.00%	975 bp	18	37.00%	875 bp
MYB83/46	24	50.00%	975 bp	20	39.50%	800 bp
Hox2a	22	45.00%	875 bp	37	63.00%	1000 bp
MYC_MYB	14	31.50%	1000 bp	8	18.50%	750 bp
MADS	13	26.00%	800 bp	8	18.50%	850 bp
LFY	9	23.50%	700 bp	4	10.50%	900 bp
ZmHox2a	5	13.00%	1000 bp	14	31.50%	900 bp
