# Supplementary material for: DNA-Demethylase Regulated Genes Show Methylation-Independent Spatiotemporal Expression Patterns
Source: Front Plant Sci. 2017 Aug 28;8:1449. doi: 10.3389/fpls.2017.01449 (PMC5581395; doi:10.3389/fpls.2017.01449)
Supplement: Supplementary file 5 [file Table_5.pdf]

Table S5: Plant *cis*-acting regulatory elements (PLACE) database results for the seven common *cis*-factors identified in DNA/HAT and RC/Helitron TE sequences that overlap with promoter regions hypermethylated in *rdd*. TE DNA sequences TAG3N1 and ATREP5 from At5G38550, SIMPLEHAT1 from At1G58602, ATREP3 5'region from At5G24210 and ATREP11 from At4G09420 (Figure 3) were submitted to PLACE (Higo et al., 1999) to identify common *cis*-factors.

***cis*-factor**	**Signal sequence**	**PLACE site #**	**Description**
ARR1AT	NGATT	S000454	"ARR1-binding element" found in Arabidopsis; ARR1 is a response regulator; N=G/A/C/T; AGATT is found in the promoter of rice non-symbiotic haemoglobin-2 (NSHB) gene (Ross et al., 2004);
CAATBOX1	CAAT	S000028	"CAAT promoter consensus sequence" found in legA gene of pea;
CACTFTPPCA1	YACT	S000449	Tetranucleotide (CACT) is a key component of Mem1 (mesophyll expression module 1) found in the cis-regulatory element in the distal region of the phosphoenolpyruvate carboxylase (ppcA1) of the C4 dicot F. trinervia; Y=T/C;
DOFCOREZM	AAAG	S000265	Core site required for binding of Dof proteins in maize (Z.m.); Dof proteins are DNA binding proteins, with presumably only one zinc finger, and are unique to plants; Four cDNAs encoding Dof proteins, Dof1, Dof2, Dof3 and PBF, have been isolated from DE maize; PBF is an endosperm specific Dof protein that binds to prolamin box; Maize Dof1 enhances transcription from the promoters of both cytosolic orthophosphate kinase (CyPPDK) and a DE non-photosynthetic PEPC gene; Maize Dof2 supressed the C4PEPC promoter;
GATABOX	GATA	S000039	"GATA box"; GATA motif in CaMV 35S promoter; Binding with ASF-2; Three GATA box repeats were found in the promoter of Petunia (P.h.) chlorophyll a/b binding protein, Cab22 gene; Required for high level, light regulated, and tissue specific expression; Conserved in the promoter of all LHCII type I Cab genes;
GT1CONSENSUS	GRWAAW	S000198	Consensus GT-1 binding site in many light-regulated genes, e.g., RBCS from many species, PHYA from oat and rice, spinach RCA and PETA, and bean CHS15; R=A/G; W=A/T; For a compilation of related GT elements and factors, see Villain et al. (1996); GT-1 can stabilize the TFIIA-TBP-DNA (TATA box) complex; The activation mechanism of GT-1 may be achieved through direct interaction between TFIIA and GT-1; Binding of GT-1-like factors to the PR-1a promoter influences the level of SA-inducible gene expression;
ROOTMOTIFTAPOX1	ATATT	S000098	Motif found both in promoters of rolD;

References:

Villain P, Mache R, Zhou DX (1996) The mechanism of GT element-mediated cell type-specific transcriptional control. J Biol Chem **271:** 32593-32598

Higo K, Ugawa Y, Iwamoto M, Korenaga T (1999) Plant cis-acting regulatory DNA elements (PLACE) database: 1999. Nucleic Acids Res **27:** 297-300

Ross EJ, Stone JM, Elowsky CG, Arredondo-Peter R, Klucas RV, Sarath G (2004) Activation of the Oryza sativa non-symbiotic haemoglobin-2 promoter by the cytokinin-regulated transcription factor, ARR1. J Exp Bot 55: 1721-1731
