# Supplementary material for: DNA-Demethylase Regulated Genes Show Methylation-Independent Spatiotemporal Expression Patterns
Source: Front Plant Sci. 2017 Aug 28;8:1449. doi: 10.3389/fpls.2017.01449 (PMC5581395; doi:10.3389/fpls.2017.01449)
Supplement: Supplementary file 8 [file Image_1.pdf]

**Figure S1**

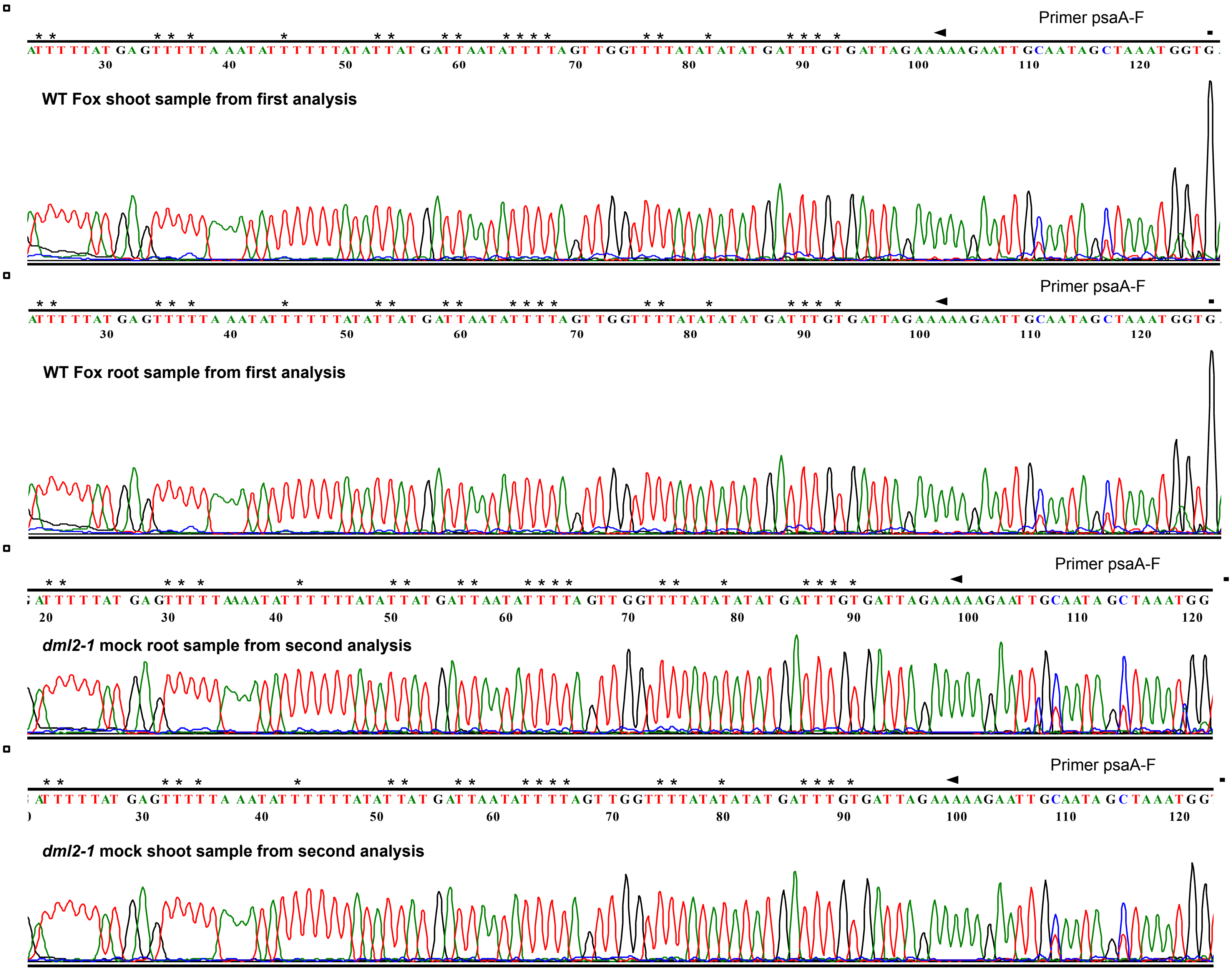


**Figure S1:** Efficiency of bisulfite conversion was 100% as determined by sequencing of the chloroplast gene *psaA*. Regions were PCR amplified from bisulfite treated DNA and the product subjected to direct sequencing. The traces from multiple reactions are shown as indicated. Asterisks indicate converted C.
