# Supplementary material for: DNA-Demethylase Regulated Genes Show Methylation-Independent Spatiotemporal Expression Patterns
Source: Front Plant Sci. 2017 Aug 28;8:1449. doi: 10.3389/fpls.2017.01449 (PMC5581395; doi:10.3389/fpls.2017.01449)
Supplement: Supplementary file 9 [file Image_2.pdf]

Figure S2

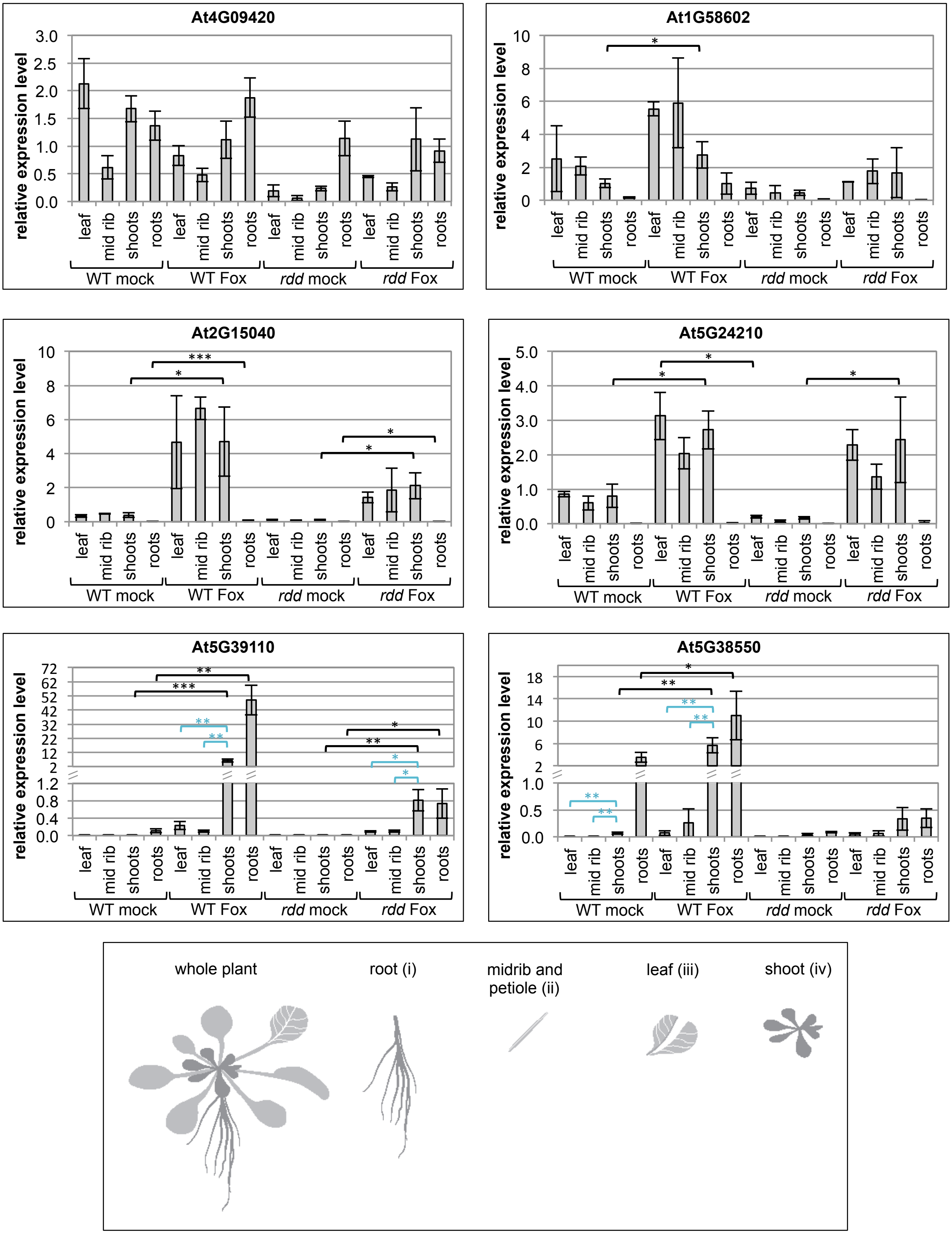

Figure S2: Expression levels of defense-related genes in WT and *rdd* tissue samples. Plants were infected with *Fusarium* and biological triplicate samples were harvested at 5dpi or 6dpi, mock or infected (Fox) respectively. Midrib and petiole sections were cut from the 5-4 largest lower leaves and the remaining leaf section as well as the remaining shoot and root sections were analysed (bottom right). Expression levels are relative to *actin2,* average is shown with error bars indicating standard deviation. Similar expression results were obtained when expression levels were compared to *FDH*. Student's *t*-tests were performed to test significance of differences in expression of shoot tissue vs. mid rib or leaf tissue (blue asterisks) as well as mock vs Fox in shoot and root tissue (black asterisks): *p≤0.05; **p≤0.005; ***p≤0.0005. Values not given are not significant.
