# Supplementary material for: DNA-Demethylase Regulated Genes Show Methylation-Independent Spatiotemporal Expression Patterns
Source: Front Plant Sci. 2017 Aug 28;8:1449. doi: 10.3389/fpls.2017.01449 (PMC5581395; doi:10.3389/fpls.2017.01449)
Supplement: Supplementary file 10 [file Image_3.pdf]

Figure S3

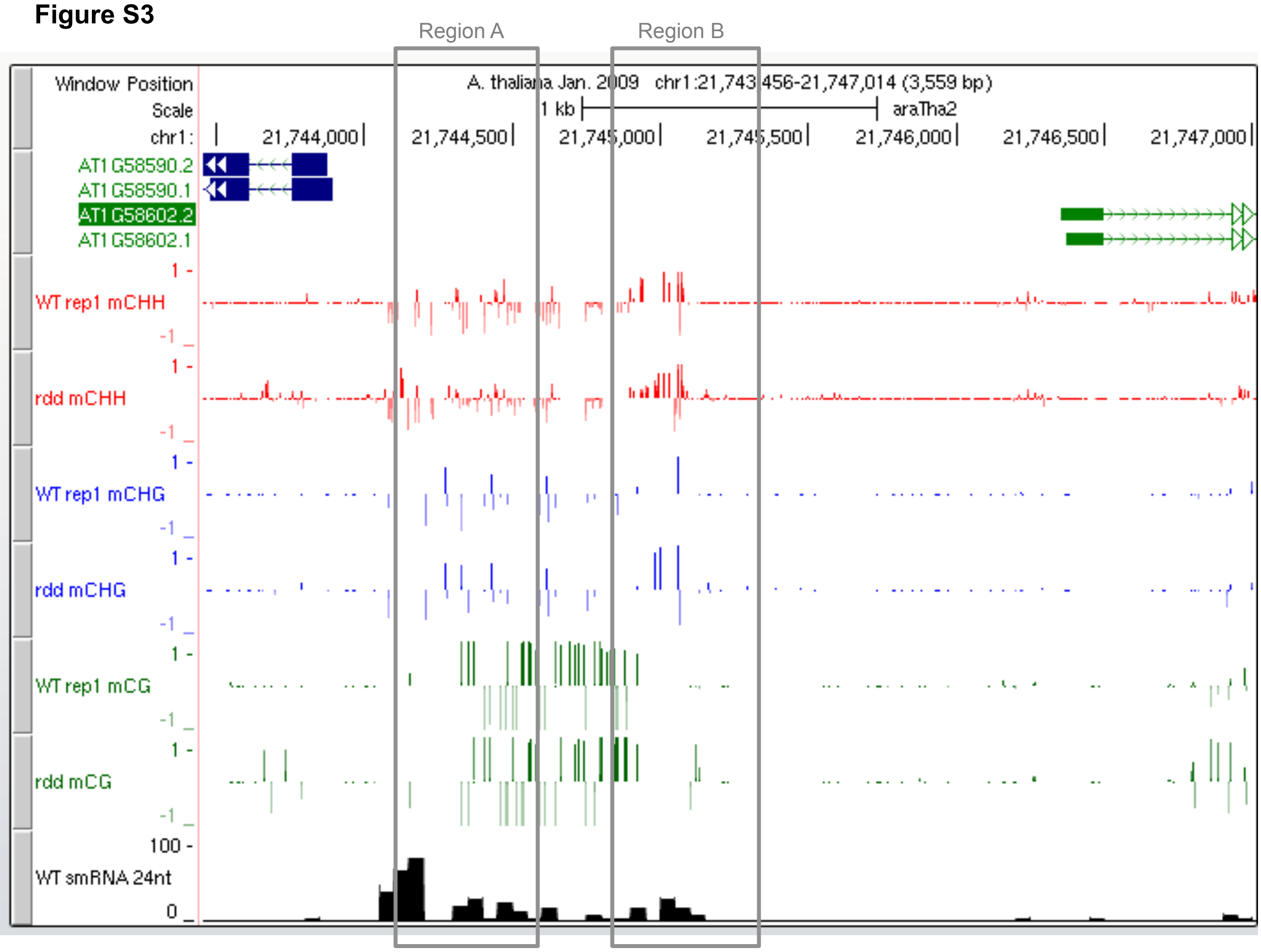

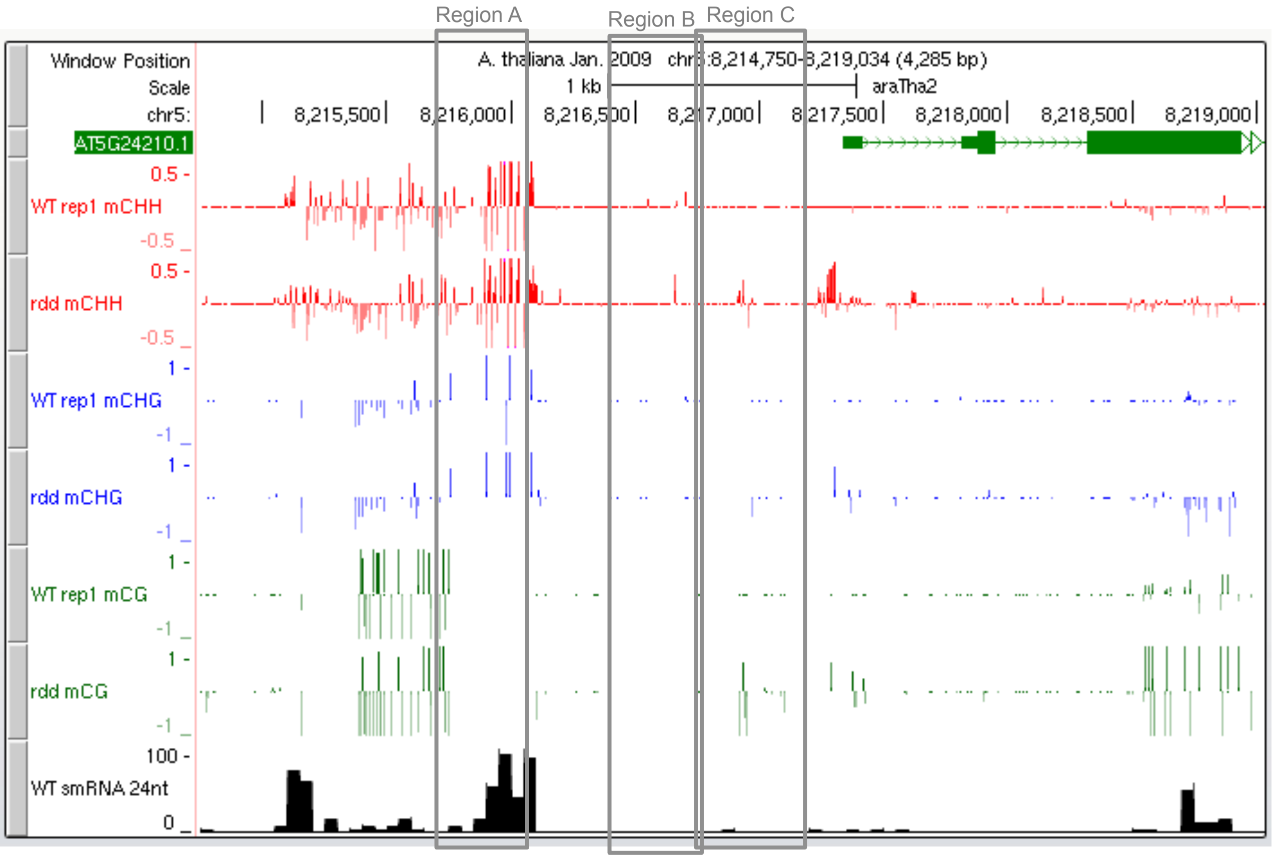

Figure S3: Screenshots of the UCSC Genome Browser (Stroud *et al.*, 2013) showing the promoter regions of defense-related genes of interest. Boxes indicate regions that have been analysed by bisulfite sequencing (Figure 3).
