# Supplementary figures and images for: DNA-Demethylase Regulated Genes Show Methylation-Independent Spatiotemporal Expression Patterns
Source: Front Plant Sci. 2017 Aug 28;8:1449. doi: 10.3389/fpls.2017.01449 (PMC5581395; doi:10.3389/fpls.2017.01449)

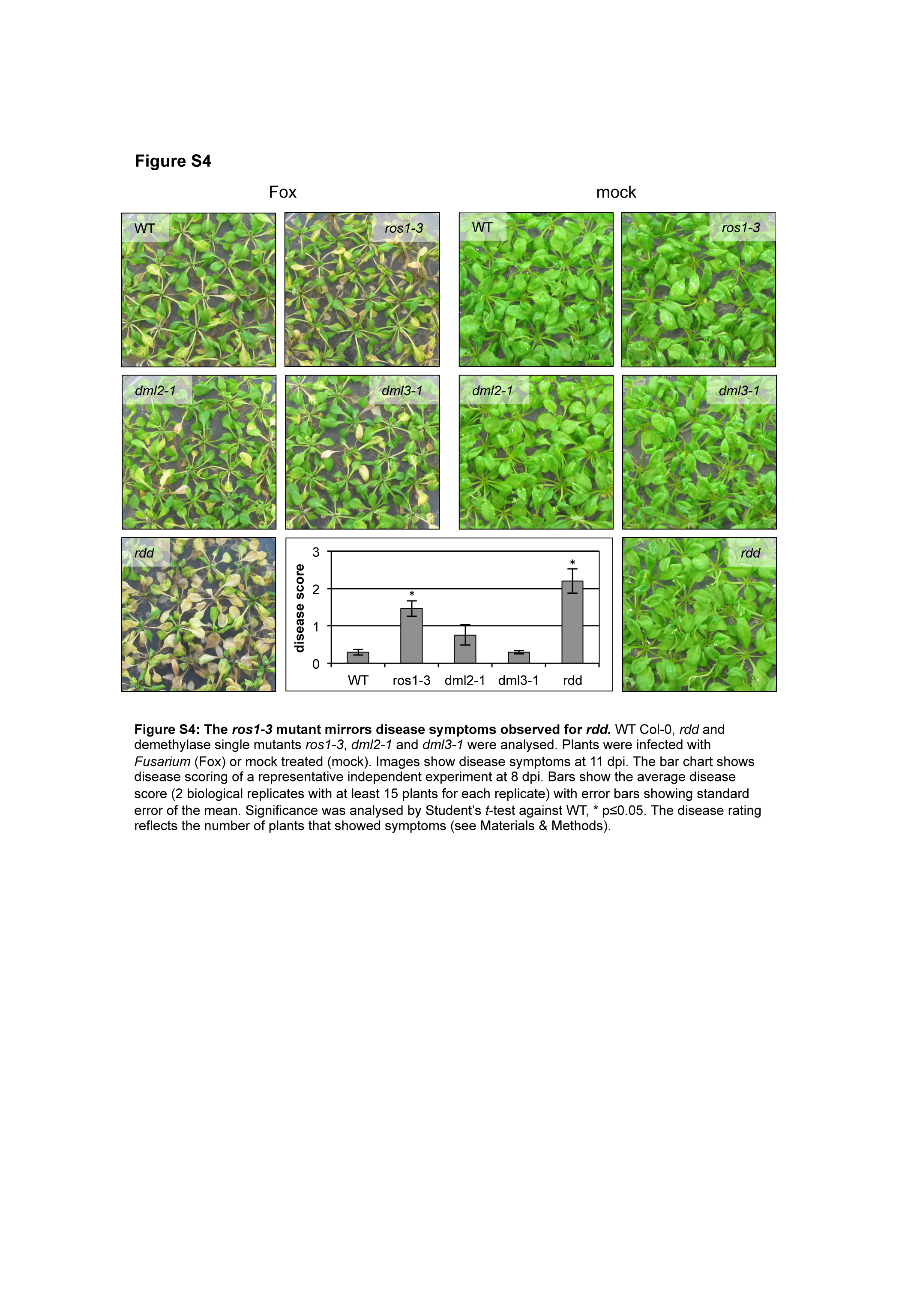

Supplement: Supplementary file 11 [file Image_4.tif]
