# Supplementary material for: DNA-Demethylase Regulated Genes Show Methylation-Independent Spatiotemporal Expression Patterns
Source: Front Plant Sci. 2017 Aug 28;8:1449. doi: 10.3389/fpls.2017.01449 (PMC5581395; doi:10.3389/fpls.2017.01449)
Supplement: Supplementary file 12 [file Image_5.pdf]

Figure S5

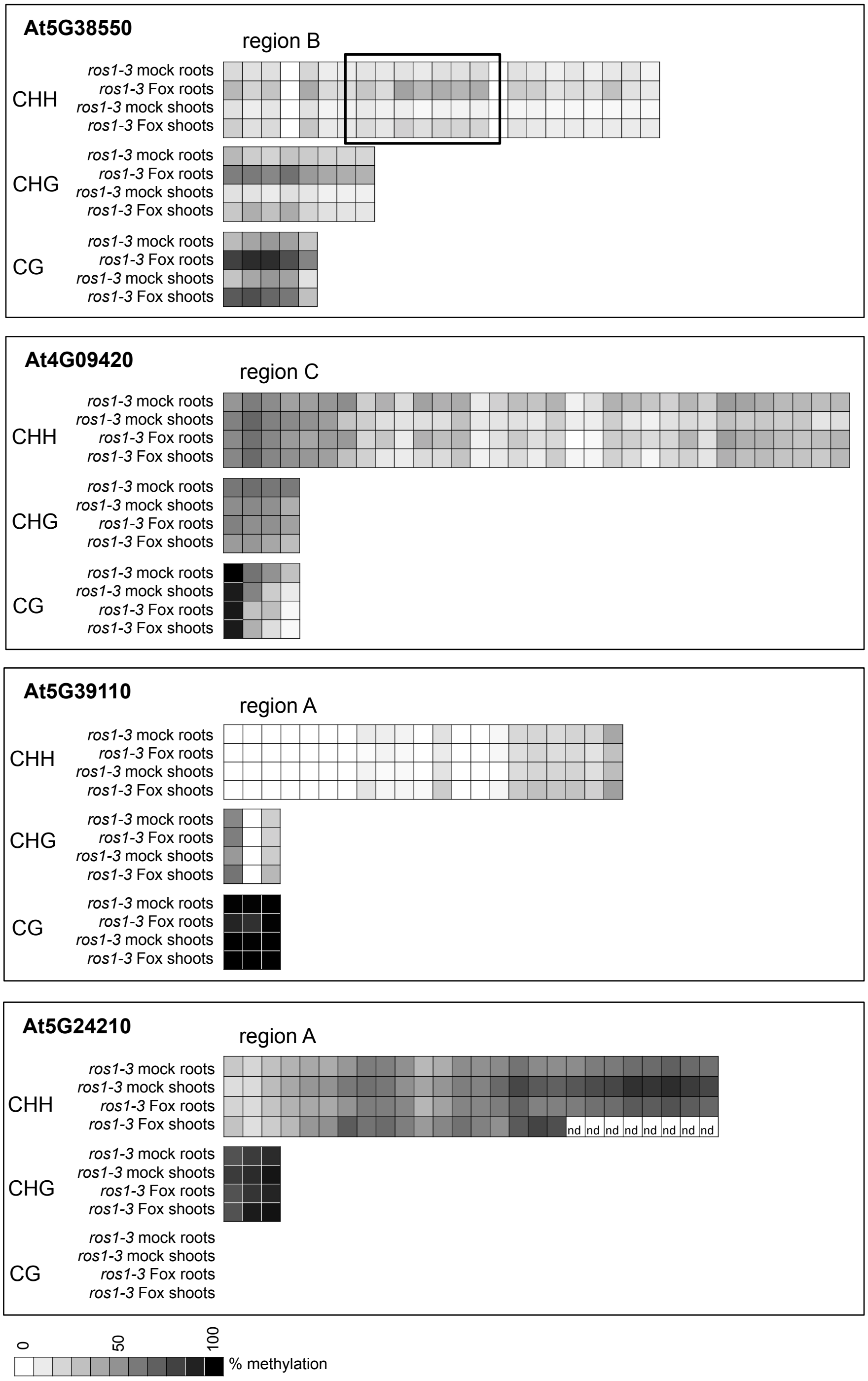

Figure S5: Tissue specific methylation differences in defense-related gene promoters in the *ros1-3* single mutant. Plants were *Fusarium*-infected or mock treated and DNA methylation analysed in roots and shoots at 3 dpi. Methylation levels were analysed using Mutant Surveyor in biological duplicates and the mean is shown. Significantly differentially methylated regions (p≤0.1) between *Fusarium*-infected and mock sample are indicated by the box. nd indicates regions for which methylation data could not be obtained. The full dataset including t-test significance values can be found in Extended Data File 2.
